# Age-dependent changes in visual sensitivity induced by moving fixation points in adduction and abduction using imo perimetry

**DOI:** 10.1038/s41598-020-78147-y

**Published:** 2020-12-03

**Authors:** Takuhei Shoji, Izumi Mine, Tomoyuki Kumagai, Akane Kosaka, Yuji Yoshikawa, Kei Shinoda

**Affiliations:** Department of Ophthalmology, Saitama Medical University, 38 Morohongo Moroyama-machi, Iruma, Saitama 350-0495 Japan

**Keywords:** Ageing, Diagnostic markers, Optic nerve diseases, Vision disorders

## Abstract

Visual field (VF) testing has usually been performed with the central gaze as a fixed point. Recent publications indicated optic nerve head deformations induced by optic nerve traction force can promote the progression of optic neuropathies, including glaucoma. We generated a new static test protocol that adds 6° adduction and abduction to gaze position (fixation points) movement. The aim of this study was to investigate both whether quantifying VF sensitivities at lateral horizontal gaze positions is feasible and whether horizontal gaze positions change sensitivities differently in subjects of different ages. Healthy adult eyes from 29 younger (≤ 45 years) and 28 elderly (> 45 years) eyes were examined in this cross-sectional study. After VF testing with central gaze as a fixation point using 24 plus (1) imo static perimetry, subjects underwent VF testing with 6° adduction and 6° abduction as fixation points. The average mean sensitivities with central gaze, adduction, and abduction were 29.9 ± 1.0, 29.9 ± 1.3, and 30.0 ± 1.2 decibels (dB) in younger subjects and 27.7 ± 1.2, 27.5 ± 1.7, and 28.1 ± 1.3 dB in elderly subjects, respectively. Visual sensitivity in young healthy subjects was similar among the three fixation points, whereas visual sensitivity in elderly healthy subjects was significantly better with abduction as a fixation point than with central gaze and adduction (both p < 0.05). We expect this test protocol to contribute to our understanding of visual function during horizontal eye gaze movement in various eye diseases.

## Introduction

Visual field (VF) testing is essential examination in the detection and management of neurological diseases. Among them, glaucoma is the world’s leading cause of irreversible blindness^1–4^. This disease causes morphologic changes such as progressive loss of retinal ganglion cells and their axons. Although mechanical stress related to intraocular pressure (IOP) is the most important risk factor for the disease, previous studies have shown that lowering the IOP does not completely suppress VF progression in every patient^5–7^. Moreover, patients with primary open angle glaucoma (POAG) who suffer glaucomatous optic nerve (ON) damage at normal IOP levels have been considered by some to have normal tension glaucoma (NTG). The Tajimi Study found the proportion of NTG to be large in Asia, particularly in Japan^6^. The NTG proportion among POAG patients in that study was 92.0%^6^. We note that IOP is not the only load that can induce optic nerve head (ONH) deformations in vivo.

A growing interest has developed in understanding whether ONH deformations induced by ON traction force can initiate the development and progression of optic neuropathies, including glaucoma^8–11^. The idea that gaze might strain the ON and surrounding eye wall dates back to Purkinje in the early nineteenth century, who suggested that traction on the ON might explain certain gaze-evoked phosphenes^12,13^. Sibony speculated that eye movements may have a role in the genesis of spontaneously acquired, asymptomatic, peripapillary subretinal hemorrhages in patients with crowded, tilted discs who otherwise were normal^14^. He provided a detailed review of this literature^10^. Renewed interest has surfaced in the effects of eye movement and gaze on the ON and in ONH deformations and their potential links to optic neuropathies^15^. Biomechanical studies of stress and strain in the posterior eye using finite element analysis have focused on the structural properties of the ONH, peripapillary sclera, and lamina cribrosa to suggest potential mechanisms for glaucoma that are independent of IOP. One novel concept is ON sheath tethering in adduction, demonstrated recently by magnetic resonance imaging (MRI)^11^. Regarding myopic eyes, Greene examined stress concentration on the posterior sclera caused by the extraocular muscles in myopia^16^. Lee et al. reported significant morphologic changes in the ONH in both abduction and adduction and these changes were associated with axial length^17^. These studies have demonstrated that eye movements can also deform the ONH, and thus may represent a pathologic process when repeated many times across a large number of everyday eye movements^11,18^.

Although recent publications have provided evidence that eye movements, horizontal duction, and horizontal gaze can significantly deform the ON sheath and ONH^8–11,15,19^, VF testing has usually been performed with the central gaze as a fixed point. Although these publications indicated structural changes in the ON due to horizontal eye movement and gaze, to date, no instrument has measured the visual sensitivity with horizontal movement as a fixation point and no study has investigated functional changes during fixation point movement. Understanding changes in a subject’s VF could be important when evaluating the effects of gaze position (fixation point) movement on both structural and functional changes. Thus, the purpose of this study is twofold: (1) to evaluate the feasibility of quantifying VF sensitivities using a new static test protocol, named horizontal gaze position (HGP) test, that adds 6° adduction and abduction gaze position movement; and (2) to investigate whether horizontal gaze change affects the sensitivities in healthy subjects of different ages.

## Results

Table 1 summarizes the ophthalmic characteristics. The mean ages of the younger (≤ 45 years) and elderly subjects (> 45 years) were 29.9 ± 6.9 years (n = 29 eyes) and 66.6 ± 10.5 years (n = 28 eyes), respectively. No subject was excluded due to unreliable VFs criteria and all subjects were eligible for analysis in all positions. No significant differences in axial length, central corneal thickness, foveal sensitivity, and mean deviation with central gaze were noted between the younger and elderly subjects’ eyes (p > 0.05 for all). The younger subjects’ eyes had better best corrected visual acuity, lower IOP, and better mean sensitivity than the elderly subjects’ eyes. Foveal sensitivity and visual field index (VFI) were not significantly different between younger and elderly eyes (Table 1). Among 15 subjects who were selected randomly, the mean deviation value for Humphrey Field analyzer (HFA) (Carl Zeiss Meditec, Dublin, CA) 24-2 of 0.1 ± 1.2 dB was comparable to that for imo central gaze of − 0.1 ± 1.2 dB (p = 0.347, paired t test).

**Table 1:** Ocular characteristics in this study protocol.

	Overall	Young adult subjects	Elderly subjects	p-value
No. of patients (n)	48	22	26	
No. of eyes (n)	57	29	28	
Age (years)	47.9 ± 20.5	29.9 ± 6.9	66.6 ± 10.5	< 0.001
Gender (male/female)	29/28	11/18	18/10	0.065
BCVA (Log MAR)	− 0.07 ± 0.04	− 0.08 ± 0.02	− 0.06 ± 0.05	0.037
Axial length (mm)	24.5 ± 1.1	24.6 ± 1.0	24.3 ± 1.1	0.370
Central cornel thickness (μm)	530 ± 33	526 ± 36	532 ± 31	0.555
IOP (mmHg)	14.7 ± 2.7	13.9 ± 2.6	15.4 ± 2.6	0.042
Foveal sensitivity (dB)	34 (31, 34)	34 (32, 34)	34 (30, 34)	0.818
Mean sensitivity (dB)	29.0 (27.6, 30.3)	30.2 (29.0, 30.8)	27.6 (26.5, 28.6)	< 0.001
Mean deviation (dB)	− 0.1 (− 1.1, 0.5)	0.2 (− 0.9, 0.6)	− 0.6 (− 1.3, 0.3)	0.104
VFI	100 (98, 100)	100 (99, 100)	99.5 (98, 100)	0.156

For normally distributed variables, the results are shown as mean ± standard deviation; for non-normally distributed variables, results are shown as median (interquartile range).

Data expressed as mean ± standard deviation were compared with a paired t-test.

Data expressed as the median (interquartile range) were compared using the nonparametric Wilcoxon rank sum test.

*BCVA* best corrected visual acuity, *SE* spherical equivalent, *IOP* intraocular pressure, *VFI* visual field index.

**Figure 1:**
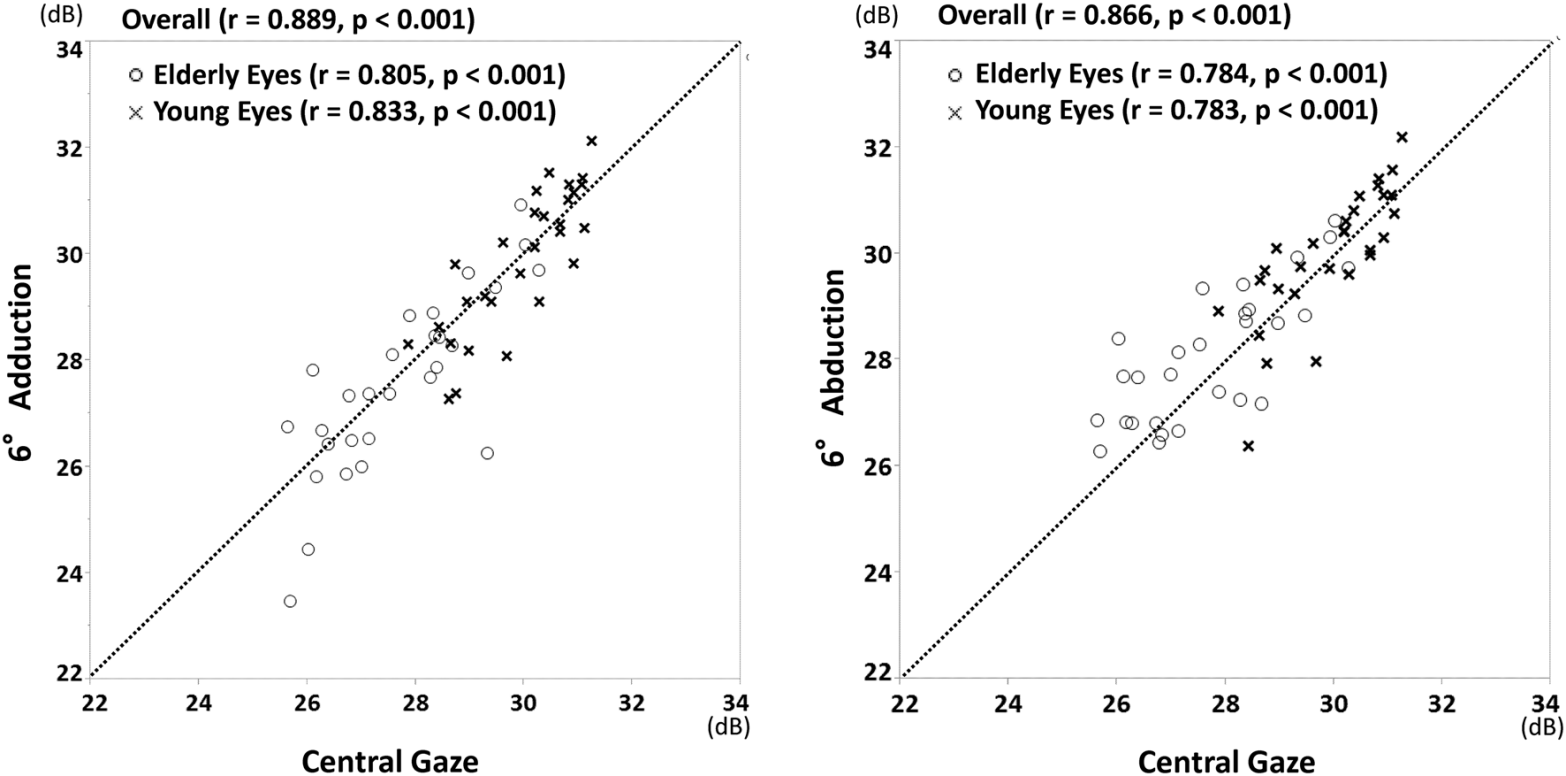
Scatterplots showing the mean sensitivity between the central gaze, 6° adduction (left), and 6° abduction (right).

**Figure 2:**
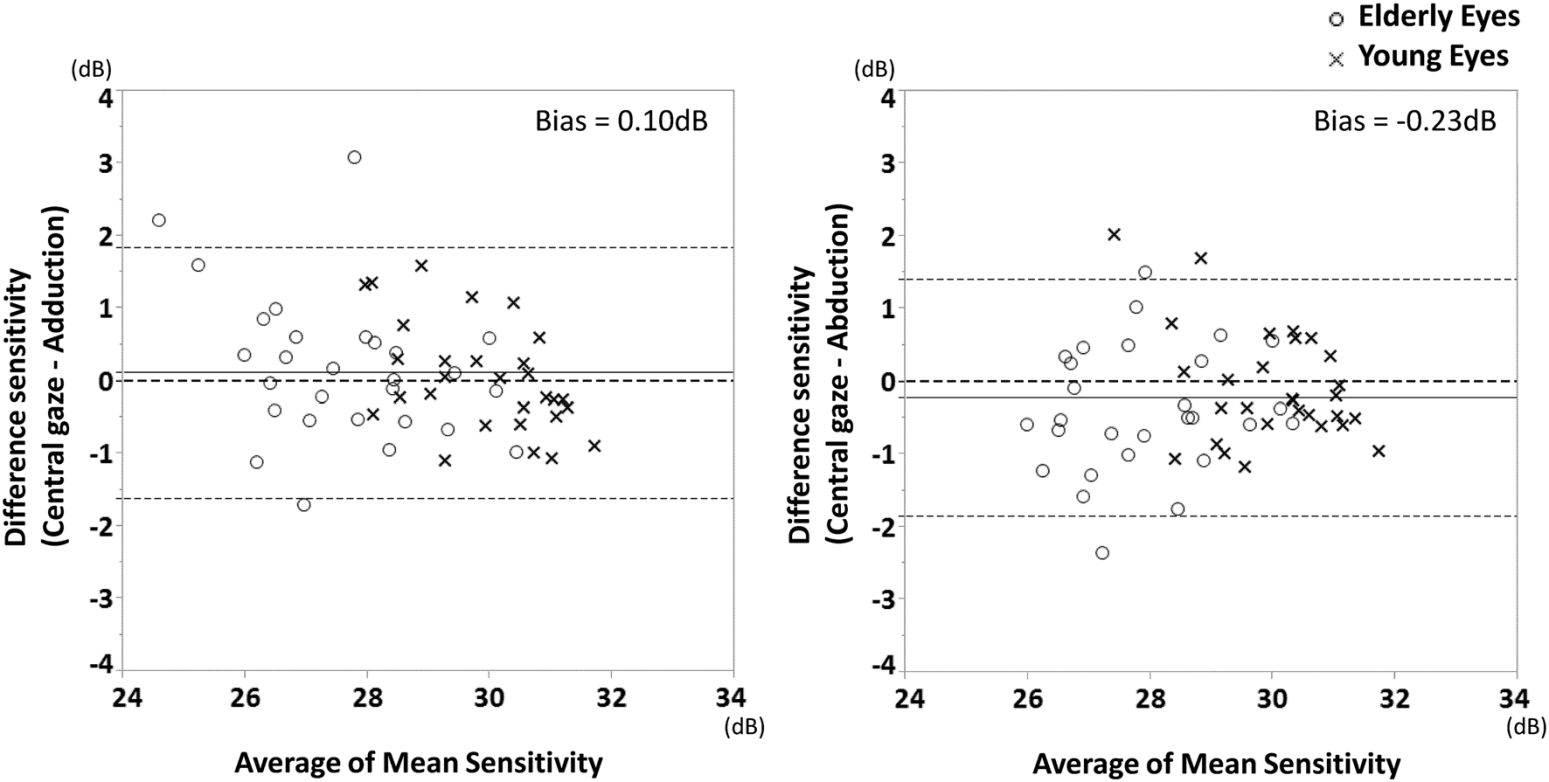
Bland–Altman plots showing the level of agreement for the mean sensitivity obtained using imo perimetry between central gaze position and abduction/adduction gaze position movement in healthy subjects. (Left) central gaze vs. 6° adduction. (Right) central gaze vs. 6° abduction. The bold dotted lines indicate the upper and lower boundaries of the 95% CIs, and the fine lines indicate the average absolute differences between the two measurements. *dB* decibels.

Figure 1 shows scatterplots comparing mean sensitivity with three different fixation positions of central gaze, adduction, and abduction. The mean sensitivity with both adduction and abduction was significantly correlated with that with central gaze both in younger and elderly eyes (all p < 0.001). Almost all of the eyes that had mean sensitivity < 28 dB were elderly. Table 2 compares the foveal sensitivity, mean sensitivity, mean deviation, and VFI among three different fixation points. Foveal sensitivity, mean deviation and VFI were not significantly different among the three positions both in younger and elderly eyes. In contrast, mean sensitivity with abduction was slightly but significantly better than either that with central gaze (mean difference of 0.4 dB, p = 0.025) or adduction (mean difference of 0.6 dB, p = 0.033) in elderly eyes. In Bland–Altman plots of all measurements, the differences were close to 0 for the mean sensitivity, which showed no systematic differences in measurement values between the two measurements (Fig. 2).

**Table 2:** Comparison the foveal sensitivity, mean sensitivity, mean deviation, and VFI among the three different fixation points.

Variables	Central gaze (CG)	Adduction (AD)	Abduction (AB)		p-value			
					CG vs. AD	CG vs. AB	AD vs. AB	
**Overall**								
Foveal sensitivity (dB)	32.4 ± 3.4	33.1 ± 2.6	33.1 ± 3.1	0.230				
Mean sensitivity (dB)	28.8 ± 1.6	28.7 ± 1.9	29.1 ± 1.6	0.024	0.384	0.042	0.020	CG = AD < AB
Mean deviation (dB)	− 0.4 ± 1.0	− 0.5 ± 1.3	− 0.3 ± 1.3	0.353				
VFI	99.0 ± 1.4	98.8 ± 2.1	99.1 ± 1.2	0.345				
**Young**								
Foveal sensitivity (dB)	32.4 ± 3.6	33.7 ± 2.3	33.9 ± 2.2	0.067				
Mean sensitivity (dB)	29.9 ± 1.0	29.9 ± 1.3	30.0 ± 1.2	0.676				
Mean deviation (dB)	− 0.2 ± 1.0	− 0.2 ± 1.3	− 0.2 ± 1.3	0.837				
VFI	99.3 ± 1.1	99.2 ± 1.2	99.2 ± 1.0	0.901				
**Elderly**								
Foveal sensitivity (dB)	32.4 ± 3.3	32.5 ± 2.9	32.4 ± 3.7	0.975				
Mean sensitivity (dB)	27.7 ± 1.4	27.5 ± 1.7	28.1 ± 1.3	0.026	0.403	0.025	0.033	CG = AD < AB
Mean deviation (dB)	− 0.6 ± 1.0	− 0.8 ± 1.3	− 0.5 ± 1.3	0.385				
VFI	98.8 ± 1.5	98.3 ± 2.6	99.0 ± 1.4	0.255				

*VFI* visual field index.

## Discussion

This study evaluated the feasibility of a new VF testing of HGP test and showed that both VF mean deviation and foveal sensitivity were similar for central gaze, 6° adduction, and 6° abduction in healthy subjects. Moreover, all measurement parameters (foveal sensitivity, mean sensitivity, mean deviation, and VFI) were comparable between central gaze position and 6° adduction among both age categories. The results indicate that our new test protocol with mild horizontal gaze position movement is similar to that with central gaze in healthy participants.

Until now, VF testing has usually been performed with the central gaze as a fixed point. In general, although both abduction and adduction can deform the ONH, the effect is greater for adduction, presumably due to ON tethering. Recent publications showed horizontal eye position strains and stretches both ON and ONH and causes morphologic changes in some ocular diseases. Using MRI, Demer found that the human ON and its sheath remain sinuous in the central gaze and in abduction, but become straightened at a threshold adduction angle beyond which these structures exert progressive tractional force on the globe^11^. A recent report by Wang et. al. indicates that (1) ONs in glaucoma subjects are tauter than those in control subjects in adduction, and (2) a tauter ON has the potential to result in earlier extinction of ON redundancy during eye movement and to cause greater stretching within the ON and ONH^20^. Lee et al. showed morphologic changes in the ONH in both abduction and adduction and these changes were associated with axial length^17^. Another study reported that deformations of the ONH^10^ and peripapillary Bruch’s membrane are greater in adduction than in abduction. Thus, we hypothesized that some affected eyes such as glaucoma and myopia would be vulnerable to ONH and the horizontal gaze position movement would significantly reduce visual function compared to control eyes. Also, if the patients have deteriorated sensitivity at the abduction or adduction position, the results would imply abnormal deterioration with horizontal eye gaze position movement. Demer et al. reported that though tethering and elongation of ON and sheath are normal in adduction, adduction is associated with abnormally great globe retraction in POAG without elevated IOP^18^. These previous studies support the hypothesis that some ocular diseases lead to a deteriorated functional index with gaze positon movement. Thus, this test protocol provides a potentially promising indicator for future study.

The effect of aging on both structural change at the ONH and the visual sensitivity change during gaze position movement has been explored but has not been fully understood. Using a scanning laser ophthalmoscope, Le et al. found that a large horizontal duction—particularly an adduction—deforms the disc and peripapillary vasculature and this displacement is greater in younger adults than in elderly adults. Thus, the ONH of young adults shifted more during both abduction and adduction than that of older subjects. Young adults have more compliant ONH tissue than elderly individuals who develop structural changes and stiffening^21^. Many past publications have also suggested that aging is associated with tissue stiffening in the lamina cribrosa^22,23^, sclera^21,24^, and Bruch’s membrane^25^. Moreover, as Le et al. speculated^19^, older adults may have less orbital fat^26^, which might enable the globe to retract more posteriorly into the orbit. This evidence may explain the difference in mean sensitivity between younger and elderly subjects and the difference in mean sensitivity between abduction and both central gaze and adduction in elderly subjects.

This study had several limitations. First, we evaluated only 6° horizontal gaze position movement, which was far less than other imaging studies that evaluated adduction and abduction of 20–35°^9,19^. However, if the horizontal gaze position was set to > 10° as a fixation point, as it was in our pilot study, subjects experienced severe fatigue and found completing the tests difficult, which resulted in low repeatability. Further study and protocol are warranted to measure the angled tolerance. Second, although we showed the mean sensitivity and mean deviation were significantly different both among positions and between age groups, the difference range was relatively small and < 1 dB. Thus, although the results may be significant statistically, they may not be meaningful clinically. This difference may not be noticed by either the practitioner or the subjects. However, the purpose of this study was to investigate whether the new test protocol can feasibly quantify the VF sensitivities in healthy subjects. Thus, future studies will determine the effect of horizontal eye gaze position movement on visual function in diseases including glaucoma, optic neuritis, high myopia^13^ and various ocular diseases. Lastly, one may argue that the small differences found might be the results of the optical instrument, rather than the observer. The different gaze positions mean that persons need to look through the optics of the head mounted display in a slightly different way or the stimuli for different gaze positions appear to be presented on different positions on the display. The VF test is a subjective test, and its reproducibility is limited. However, an important finding in this study is that the results in this test mode (6° abduction and adduction as fixed point) were about the same as central gaze, at least in healthy young subjects. Even in elderly subjects, the sensitivity in adduction was similar and very close to central gaze. We believe that this is an important finding for future research on diseased eyes.

In conclusion, we have generated a new VF measurements protocol with horizontal eye gaze position movement as a fixation point, named the HGP test, and shown that these sensitivities are feasible, and produced comparable results of foveal sensitivity and VFI scores in both young and elderly healthy eyes. Conversely, mean sensitivity with abduction as a fixation point was slightly but significantly better than with central gaze and adduction in elderly healthy subjects. We expect this test protocol to contribute to our further understanding of visual function during horizontal eye gaze position movement in various eye diseases, particularly glaucoma and high myopia.

## Materials and methods

### Study population

This cross-sectional study was approved by the Ethics Committee of Saitama Medical University (No. 20118.01) and was conducted in accordance with the tenets of the Declaration of Helsinki. Healthy volunteers admitted between October 2017 and September 2020 were included if they were ≥ 20 years old, fulfilled the eligibility criteria detailed below, and provided informed consent.

All subjects underwent a comprehensive ophthalmic examination, including slit-lamp biomicroscopy and IOP measurement by noncontact tonometry (Tonoref II; Nidek Co., Ltd., Aichi, Japan). Axial length and central corneal thickness were also measured (Optical Biometer OA-2000, Tomey Corp., Nagoya, Japan). Eyes were excluded if any of the following were true: best corrected visual acuity worse than 20/40, spherical refractive error worse than − 8.0 or + 3.0 diopter, cylinder refractive error worse than 3.0 D, and axial length > 26.5 mm or < 22.0 mm. Participants with a history of intraocular surgery (except for uncomplicated cataract surgery), coexisting retinal pathologies, non-glaucomatous optic neuropathy, uveitis, ocular trauma, strabismus or fusion disorder, or history of Parkinson’s disease, Alzheimer’s disease, dementia, or stroke were also excluded^27^.

### Imo perimetry

**Figure 3:**
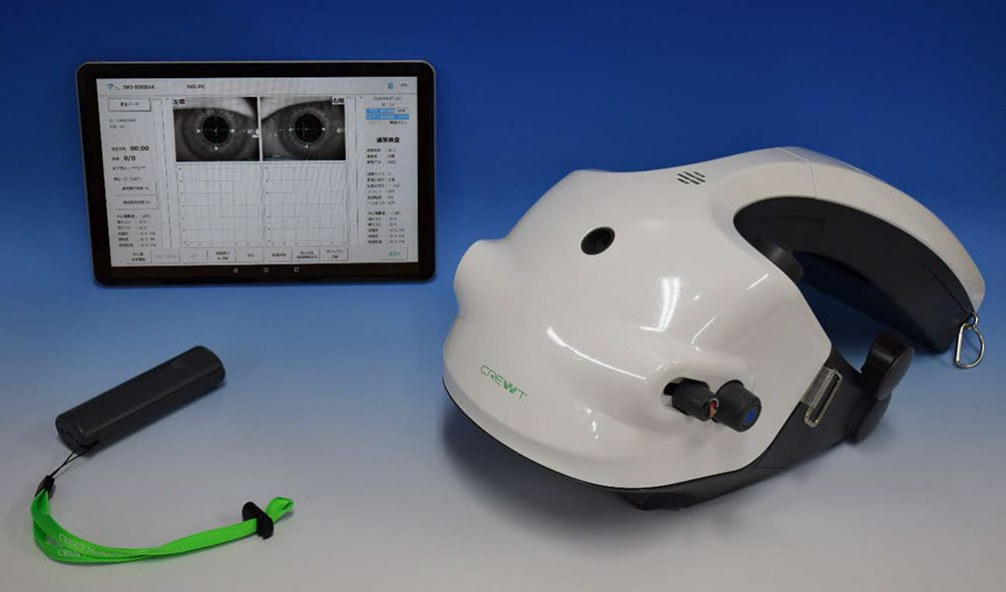
The new static perimeter imo consists of a main perimeter unit, a user control tablet, and a subject response button.

**Figure 4:**
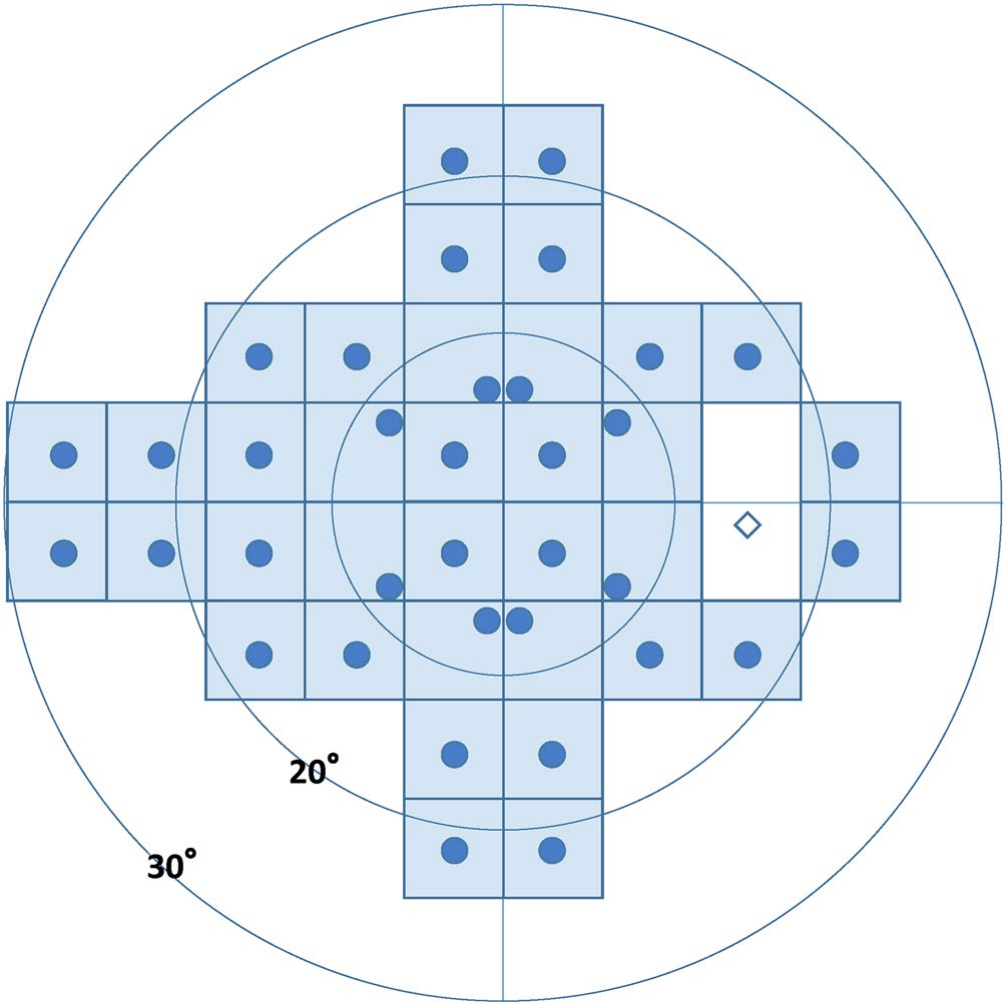
Schematic representation of the test locations (right eye), in which 24plus (1) extracts 36 points along the nerve fiber layer near the fixation point of 24-2.

We tested VF testing with different gaze conditions using the imo perimeter (Fig. 3) as described previously^28–31^. Briefly, during an imo test, the target is presented to either eye using a full high-definition transmissive liquid crystal display and is backlit with a high-intensity light-emitting diode. The maximum target luminance is 3183 cd/m^2^ (10,000 asb) and the background luminance is 10 cd/m^2^ (31.4 asb). The imo is equipped with two separate sets of optical systems and pupil-monitoring systems for right and left eyes. Using an SXVGA-resolution (1280 × 960 pixels) complementary metal-oxide semiconductor sensor with a maximum frame rate of 54 Hz, images can be recorded in real time. In this study, 36 points in total located within the central 30° VF were tested using Goldmann size III (0.431° visual angle) stimuli. The threshold algorithm used a 4–2-dB bracketing strategy to perform the central gaze test. It has been shown that the VF sensitivity of imo under central gaze position was highly compatible to that obtained by standard automated perimeter using HFA^28,32^. After measuring the sensitivity of the central gaze as a fixation point, additional positions at both 6° abduction and 6° adduction as fixation points (Fig. 4) were measured using Ex-mode. The order of these two positions was assigned randomly. Detailed methods are described below.

### 24plus (1) and Ex-mode

**Figure 5:**
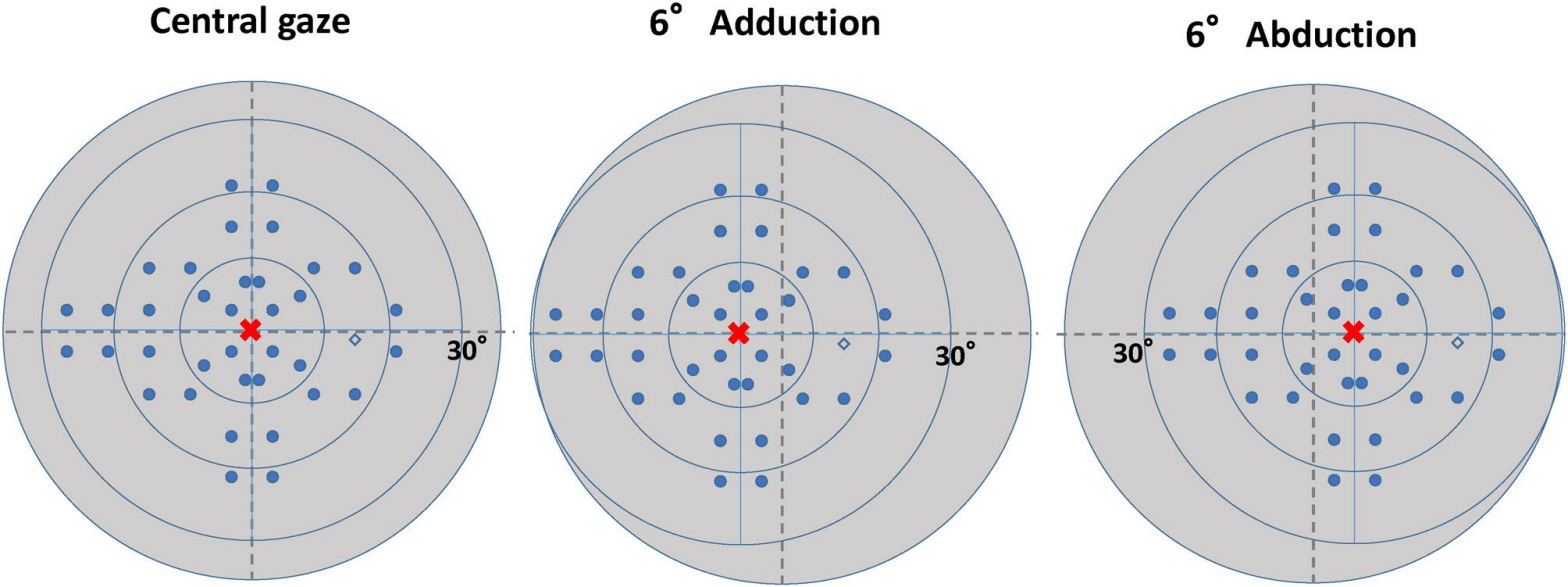
Schematic explanation of the central gaze with 6° adduction and 6° abduction as fixation points (right eye). Visual field (VF) testing was performed with the central gaze (left) as a fixation point. This testing is common in the currently available static perimetry. VF testing was performed with 6° adduction (center) and 6° abduction (right) as fixation points. Each test point was also moved based on the fixation point.

To reduce testing time and subjects’ fatigue, we used both 24plus (1) and Ex-mode. The 24plus (1) mode consists of 36 test points that are selected as likely places to detect early glaucomatous VF changes^32^. These test points result from combining the 24-2 and 10-2 test modes. Figure 5 shows the test points in detail. The Ex-mode considers the threshold data for each subject in the past and enables improved inspection accuracy and reduced test time. In this study, Ex-mode was used for adduction and abduction tests based on the data of the central gaze. Subjects were assigned randomly to either direction test first.

Unreliable VFs, defined as fixation losses > 25% or false-positive responses > 15%, were excluded. Mean sensitivity was calculated in dB using individual test points, where each point was converted to a linear scale (1/Lambert = 10^0.1XdB^; linear sensitivity)^33–35^ and averaged to obtain the mean sensitivity values.

### Statistical analysis

The distribution of numerical variables was assessed by using the Shapiro–Wilk W test of normality. The results of non-normally distributed variables are shown as median (interquartile range) and normally distributed variables are shown as mean ± standard deviation. Wilcoxon rank sum tests were used to compare the variables between young and elderly eyes. The repeated measures analysis of variance (ANOVA) and followed by post hoc Bonferroni test for multiple comparisons was used to evaluate foveal sensitivity, mean sensitivity, mean deviation and VFI. The agreement between two measurements was evaluated using Bland–Altman plots. All statistical analyses were performed using JMP version 11 (SAS Institute Inc., Cary, NC) and Stata version 15 (StataCorp LP, College Station, TX) and any p-value < 0.05 was considered statistically significant. To assess the reliability of the measurements of imo and HFA visual sensitivity, we compared HFA24-2 and imo central gaze position for subjects (n = 15) who were selected randomly.

## Data Availability

The datasets generated and/or analyzed during the current study are available from the corresponding author upon reasonable request.
